# Ecological restoration in a cultural landscape: conservationist and Chagossian approaches to controlling the ‘coconut chaos’ on the Chagos Archipelago

**DOI:** 10.1007/s10745-014-9696-y

**Published:** 2014-08-23

**Authors:** Laura Rebecca Jeffery

**Affiliations:** University of Edinburgh, Edinburgh, UK

## Restoration Ecology and Native, Non-native, and Invasive Species

Restoration ecology can broadly be defined as the theory and practice of attempting to reverse anthropogenic damage to natural ecosystems (cf. Higgs 1997: 340–342; Jackson *et al.* 1995). Activities undertaken in pursuit of ecological restoration include the removal of invasive species to improve conditions for native species. A native or indigenous species is considered to belong naturally to a certain area, having evolved in that area or been transferred there by ‘natural forces’ such as animals, water, or wind (Beinart and Middleton 2004: 15). An alien or exotic species is considered not to belong in a certain area, and to have been transferred there intentionally or accidentally as a result of human activity; it is seen as ‘matter out of place’ (Milton 2000; Peace 2009). An invasive species is one whose spread threatens biodiversity, economic security, or human health (Simberloff 2003: 189).

Definitive classifications of species as native, alien, or invasive, however, are complicated. There may be geographical, spatial, temporal, logical, or evidential challenges to identifying a species according to its natural range (Head and Muir 2004: 201–202; Warren 2007: 431–432; Woods and Moriarty 2001: 176). Geographically, to what extent is the concept of a natural range influenced by non-natural, historical, national boundaries (Olwig 2003)? Spatially, what is the relevant scale, and what are the implications of variation within a supposed site (Warren 2007: 432)? Temporally, how can determination of the relevant historical period avoid being arbitrary (Warren 2007: 431)? Logically, how should one classify a species if it was once present in an area, wiped out prior to human habitation, then reintroduced by humans (Kendle and Rose 2000: 20; Warren 2007: 432)? Evidentially, what about a species that could have been established in an area either through human agency or in the absence of human activity (Kendle and Rose 2000: 21)? Rather than seeing an immutable boundary, Woods and Moriarty (2001: 174–176) instead propose native and exotic as ‘cluster concepts’ identifiable along a continuum according to several characteristics: whether or not humans were responsible for the presence of the species in an area; the location of the original evolution of the species; the historical (or natural) range of the species; the impact of the species on the local ecosystem; and the degree to which the species is integrated in the ecological community. This allows for definitions of species as more or less native than other species, and for the recognition of changes over time such that a species may adapt or evolve in a process of ‘naturalisation’ in its new habitat from alien to native (Woods 2999: 176).

Secondary to classification along the native/alien axis, ecologists classify species according to their function, behaviour, and effects on other (native) species (Kendle and Rose 2000: 22). The US National Invasive Species Council defines invasive as ‘an alien species whose introduction does or is likely to cause economic or environmental harm or harm to human health’ (quoted in Simberloff 2003: 189). As this implies, invasive is usually taken to be a subcategory of alien (Woods and Moriarty 2001: 170). Indeed, alien species may be more prone to becoming invasive because of an absence of natural predators (Kendle and Rose 2000: 24), although only an estimated 0.1 % of aliens become invasive (Williamson 1996). Native species can also become invasive (Head and Muir 2004: 199), but an analysis of hundreds of articles about invasive plants in the United States concluded that alien species were 40 times more likely than native species to be perceived as invasive (Simberloff *et al.* 2012: 600). However, even an invasive species might simultaneously harm and benefit a natural ecosystem by displacing some native species while supporting other native species (Woods and Moriarty 2001: 171). Consequently, the removal of invasive species might facilitate invasion by other more aggressive alien species and may harm native wildlife that utilises alien hosts (Woods 2999: 183).

The classification of a species as invasive/non-invasive may also depend on its perceived social utility measured in terms of economic value, cultural significance, or aesthetic attributes (Helmreich 2005: 111–112; Warren 2007: 430; Woods and Moriarty 2001: 181). But this rationale is also problematic, since classifications of desirable/undesirable species vary (Beinart and Middleton 2004: 16–18; Kendle and Rose 2000: 24; Milton 2000: 230–231), and ‘exotic introductions are often woven into people’s sense of place,’ providing food and livelihoods (Warren 2007: 434). Conservation activities take place in landscapes with ecological, economic, political, social, and/or cultural significance, and are often fraught with tensions between competing stakeholders such as conservationists and local land users (Comaroff and Comaroff 2001: 637–644; Peace 2009: 59, 62; Trigger *et al*. 2010). Offering an anthropological critique of biodiversity discourses, I explore the tensions in an ecological restoration project on the remote Indian Ocean island of Diego Garcia in the Chagos Archipelago, where the species classified by conservationists as invasive has economic, historical, and socio-cultural significance for the displaced former inhabitants of the islands.

## A Brief History of the Chagos Archipelago

The Chagos Archipelago is a group of coral atolls and islands in the middle of the Indian Ocean. The Chagos islands were unpopulated by humans prior to European colonial expansion from the late eighteenth century onwards, when French planters brought enslaved labourers mostly from mainland East Africa and Madagascar via Mauritius. The Colony of Mauritius and its dependencies – including Chagos – transferred to British control during the Napoleonic Wars. Throughout the settled history of the Chagos islands, coconut plantations were the economic base and main source of employment. Also known as the Oil Islands, they relied almost entirely on the production of copra (dried coconut flesh) exported for the extraction of coconut oil that was refined for energy and used in the production of soap (among other products) in Mauritius and beyond.

In 1965, the UK Government excised Chagos from Mauritius to form part of the new British Indian Ocean Territory (BIOT) before granting independence to Mauritius in 1968. In 1966, the UK Government made the largest Chagos island of Diego Garcia available to the US Government for an overseas military base. Successive Mauritian governments have claimed sovereignty of the Chagos Archipelago, but the UK’s response is that Chagos will be returned to Mauritian sovereignty when it is no longer required for defence purposes. The UK Government depopulated the Chagos islands by 1973. Over this period as a whole, between 1,328 and 1,522 islanders ended up in Mauritius and 232 in Seychelles (Gifford and Dunne 2014: 46). Chagos islanders and most of their second-generation descendants were awarded UK citizenship under the British Overseas Territories Act in 2002, since when up to 2,000 of the extended Chagossian community have moved to the UK, although by far the largest concentration, totalling around 700 surviving Chagos islanders and several thousand of their descendants, still live in Mauritius (Jeffery 2013: 303).

Since their displacement, Chagossian groups in exile have campaigned for compensation and the right of return to Chagos. Chagossians in Mauritius (but not Seychelles) received some compensation in the late 1970s and early 1980s (Jeffery 2011: 27–28), but a Chagossian coalition’s legal application for further compensation was rejected in the English Court of Appeal in 2004 (Jeffery 2011: 41). In 2000, the High Court declared that their removal had been unlawful, but in 2004 the UK Government used a prerogative Order in Council to overturn this decision and ban the Chagossians from returning (Jeffery 2011: 129–132). The UK Government’s longstanding opposition to resettlement on the grounds of feasibility, cost, and military security has been joined in the past decade or so by environmental arguments based on the ecological importance of the Chagos Archipelago. In 2010, responding to a campaign led by a coalition of conservation groups, the UK Government declared a no-take (i.e., no fishing) Marine Protected Area (MPA) around the Chagos Archipelago (see Jeffery 2013: 302–304). Olivier Bancoult, the leader of the Chagos Refugees Group (CRG), applied for judicial review of the MPA on the grounds inter alia that it was established with the “improper motive” of preventing the islanders from returning to the territory, but both the High Court and the Court of Appeal ruled in favour of the UK Government (see Jeffery 2014). The Mauritian Government is contesting the MPA under the UN Convention on the Law of the Sea (UNCLOS) on the grounds that the UK Government does not have the jurisdiction to declare an MPA in this disputed territory. Meanwhile, in 2013–2014 the UK Foreign and Commonwealth Office (FCO) consulted the Chagossian communities and other stakeholders in Mauritius and the UK and commissioned a new feasibility study on resettlement.

Here I compare conservationist and Chagossian approaches to controlling the overgrown coconut plantations on Diego Garcia. Conservationists classify *Cocos nucifera* as an alien invasive species detrimental to biodiversity, while Chagossians value the coconut plantations as part of a cultural landscape that once provided employment, food, and artisanal products. Following calls by social scientists for the integration of natural and cultural heritage conservation (Harmon 2007; Lowenthal 2005), I suggest that the conclusions drawn by conservationists and Chagossians respectively have more in common than is usually supposed, and I conclude that the two groups could together coordinate ecological restoration alongside restoration of the cultural landscape.

I have conducted periods of participant observation with the displaced Chagossian communities since 2002. Fieldwork in Mauritius and the UK from 2011–2013 focused on debates about environmental knowledge and included semi-structured interviews and conversations with Chagossians, academic ecologists, conservationists associated with environmental NGOs, independent environmental consultants, fisheries businesspeople, former administrators of Chagos, diplomats, civil servants, politicians, political activists, lawyers, social scientists, and members of Chagossian support groups. I ran a series of focus groups with Chagos islanders and their descendants, and I assisted at the FCO’s consultations with Chagossian groups. In addition to material from many conferences and workshops focusing on Chagos, including several of the annual Chagos Conservation Trust (CCT) scientific conferences since 2002, I also draw on scientific publications and contributions to the Chagos Conservation Trust (CCT) newsletter by ecologists and conservationists with field experience in the Chagos Archipelago.

## Native, Alien, and Invasive Species in the Chagos Archipelago

The Chagos Archipelago comprises small coral islands the Indian Ocean with a total land area of only around 56.13 km^2^, of which Diego Garcia constitutes 32.50 km^2^. The 200 nautical-mile Exclusive Economic Zone (EEZ) extends to 640,000 km^2^. The MPA covers the whole of the EEZ except 470 km^2^ comprising the coral atoll of Diego Garcia with its lagoon and three nautical mile territorial waters (Sand 2010: 232). Scientific data about Chagos come from periodic British-led scientific research expeditions since the nineteenth century and from surveys of Diego Garcia carried out by the US military since the early 1970s for the purposes of planning natural resource management. The Indian Ocean around Chagos is an area of high abundance and biodiversity of corals and fish, and is home to at least 10 known endemic species including the Chagos brain coral and the Chagos clownfish. Natural scientists agree that the marine environment is more exceptional than the terrestrial environment in terms of biodiversity, endemism, genetic interconnectivity with the Western Indian Ocean, and the apparent absence of alien invasive species (Sheppard *et al*. 2012).

The Chagos islands harbour no endemic terrestrial flora, but perhaps three endemic subspecies of butterfly and one endemic species of moth (Barnett and Emms 1999: 243), and have also become an important haven for native fauna. Chagos islanders ate coconcxut crabs and the meat and eggs of green sea turtles and a range of ground-nesting birds, and green sea turtles and hawksbill turtles were exported to Mauritius for their meat and shells respectively (Frazier 1980: 343). Chagos scientists have reported that such practices resulted in diminished populations of these species by the middle of the twentieth century, but that – four decades after the depopulation of the islands – monitoring and the deterrence of poaching in the vicinity of the US military base have resulted in some of the world’s largest concentrations of hawksbill turtle, red-footed booby, and coconut crab at various sites on Diego Garcia (Sheppard *et al*. 2012).

There are no native mammals, but several mammal species have been introduced over the course of the settled history of the islands, with rats, cats, and donkeys causing the most consternation. Cats and rats eat turtle eggs and hatchlings and ground-nesting seabirds, eggs, and chicks. Plantation owners evidently periodically offered financial incentives to Chagos islanders for every rat carcass produced (Wiehe 1939: 23; Stoddart 1971: 216). In the 1990s, conservationists advocated rat eradication to assist the conservation of sea turtles and seabirds (Mortimer and Day 1999: 170; Symens 1999: 271), but a 2006 rat eradication programme on Eagle Island failed (Meier 2006: 2–4; Topp 2007: 1). In 2005, the US Navy reported on rat and cat control programmes at the military base on Diego Garcia, and proposed extending rat eradication measures to the entire island (US Naval Facilities Engineering 260 Command (Pacific Division) 2005: Appendix I-2). Donkeys had been imported by the 1840s as haulers in the coconut plantations, but were released upon mechanisation in 1938, after which the population grew (Stoddart 1971: 215). Donkeys eat native vegetation, apparently favouring *Pisonia grandis*, a hardwood that provides nesting sites for breeding seabirds (Carr 2011: 8–9). In the early 1970s the US Navy advocated eradication on the grounds that donkeys threatened vegetation and were a potential hazard to landing aircraft (US Naval Facilities Engineering Command (Pacific Division) 1973: 16–19). In 2005, the US Navy again proposed donkey eradication or contraception (US Naval Facilities Engineering Command (Pacific Division) 2005: Appendix H-2).

Scientific research on the Chagos islands has recorded 232 alien terrestrial flora species, of which 128 occur only on the island of Diego Garcia (Clubbe 2010: 8), which has been exposed to the majority of human traffic throughout the settled history of the archipelago but especially since the establishment of the US military base and the depopulation of the outer islands. A botanical survey of Diego Garcia carried out for the US Navy in 1995 categorised alien plant species as either ‘weedy’ (accidentally introduced) or ‘cultivated’ (deliberately introduced): 102 ‘weedy’ species had previously been recorded, of which 16 were no longer found in 1995, but 32 new species had been introduced during the previous decade; 96 ‘cultivated’ species had previously been recorded, of which 26 were no longer found, but 29 new species were recorded for the first time (Whistler 1996: 5–6). Many of the ‘cultivated’ species that had disappeared were food crops, including squash and sweet potato, that had been cultivated by the Chagos islanders. The explanation given for the appearance of new cultivated species was that ‘contract workers and military personnel commonly bring in, officially or surreptitiously, new ornamental or crop plants to cater to their culinary or aesthetic desires’ (Whistler 1996: 6). Whistler recorded 12 native tree species, including coconut palms, but noted that ‘some of these may have been ancient or early introductions that are now naturalized’ (Whistler 1996: 3). Trees including *Cocos nucifera* and *Casuarina equisetifolia* apparently threaten the (re) growth of hardwoods (such as *Baringtonia asiatica* and *Pisonia grandis*) that provide nesting sites to breeding seabirds (Sheppard *et al.* 2012).

## Spatial Challenges in Classifying Coconut Palms as Native or Alien

Reports of voyages to the Chagos Archipelago since its discovery indicate the presence of coconut palms (*Cocos nucifera*) long before the islands were settled or commercially exploited by humans. John Davis, a navigator, and Edward Michelbourne, a knight, led an expedition to Chagos on the *Tigre* in 1605, almost two centuries before French planters established the coconut plantations. An account of this voyage reported of Diego Garcia that:

This Iland seemeth to bee some ten or twelve leagues long, abounding with Birds and Fish; and all the Iland over seemeth to be a mightie Wood, of nothing else but Coco-trees. What else this Iland yeeldeth we know not. (Anon 1880: 167)

When an expedition led by Captain Lazare Picault visited Peros Banhos Atoll in 1744, five of the islands were apparently already covered in coconuts (Wiehe 1939: 3–4). Sailing past the unpopulated Three Brothers in 1771, Captain J.M.C. du Roslan reported that they were ‘covered with very lofty cocoa-trees and other wood of inferior height’ (quoted in Scott 1961: 69), an observation corroborated after a visit in 1786 by the hydrographer Archibald Blair, who reported that two of the Three Brothers were ‘abounding with *Coconut Trees*’ (Blair 1788: 17). Blair noted that the Six Isles (also known as Egmont Atoll) were low and covered with wood; three of them ‘only abound with *Coconut Trees*’ (Blair 1788: 16). He described Danger Island as being ‘covered with thick Wood, and a few *Coconut Trees* near the Centre’ (Blair 1788: 17). The larger of the two Eagle Islands was also ‘covered with *Coconut Trees*’ (Blair 1788: 17).

The first attempts to cultivate Diego Garcia in 1784 were unsuccessful, but exploratory settlement and commercial exploitation were underway within a couple of years (Wiehe 1939: 3; Scott 1961: 20, 75–76). Production on Diego Garcia and Egmont Atoll was expanded from 1808 onwards (Scott 1961: 96–101, 260), and other islands in the archipelago were settled and/or commercially exploited from the early nineteenth century onwards: Peros Banhos Atoll, the Three Brothers, and Eagle Island from 1813, and Salomon Atoll from around 1817 (Wiehe 1939: 4; Scott 1961: 19, 119, 266, 276). Gilbert Bourne, a zoologist who visited the coconut plantations on Diego Garcia in 1885, reported that:

Properly speaking, there is no cultivation of the coco-nut; the palms have existed on the island from time immemorial, and sow themselves, only in a few places have they been artificially planted. (Bourne 1888: 388)

In 1939, P.O. Wiehe, a plant pathologist, reported that environmental conditions were ‘extremely favourable’ to the development of coconuts, which could be found on all the islands as a result of human introduction (Wiehe 1939: 11). He noted that ‘regular plantations’ were only found where soil conditions were ‘more unfavourable’ (Wiehe 1939: 11), particularly in Peros Banhos Atoll (Wiehe 1939: 15). He considered the majority of groves to be ‘natural,’ by which he meant that they ‘owe their origin to a regular plantation in the past, but regeneration of seedlings, the development of various trees and a dense undergrowth confer to them the aspect of a jungle’ (Wiehe 1939: 13).

Robert Scott (1961), then Governor of Mauritius, pointed out that:

There have evidently been cyclic changes … in the capacity of coral islands to bear heavy groves of palms. When Captain Picault visited Peros Banhos in 1744, very few of the islands in the group carried coconuts. According to records of a decade later some of the islands were ‘found covered with cocoa-trees’. As had been established in Agalega – and the point could still be demonstrated in Peros Banhos even in the thirties of this century – a survey on foot would show that impressions of luxuriant growth gained from shipboard could be unduly flattering. Thick belts of palm would be formed round the coasts, but the interior of the islands might have only a sparse covering of vegetation… It has been held by more modern investigators also that, whereas the introduction of the palm to the Lesser Dependencies was by way of ocean currents, the extensive growth of coconuts throughout the islands has been the result of human agency. In some of the islands, however, the natural growth must have been very impressive. (1961: 19)

In their report following the 1996 scientific expedition to Chagos, Commander John Topp and ecologist Charles Sheppard included *Cocos nucifera* on a list of ‘probable natives’ on 47 of the Chagos islands (Topp and Sheppard 1999: 236), remarking that coconut seeds ‘can travel 6,000 km and survive eight months at sea,’ and it is therefore likely that coconuts ‘came naturally by sea’ to Chagos (Topp and Sheppard 1999: 233). However, they implicitly contrasted the coconuts with the ‘original native trees’ that have made a comeback in some places since the abandonment of the coconut plantations. In the following chapter, zoologists Linda Barnett and Craig Emms reclassified *Cocos nucifera* as ‘non-native … dominant introduced vegetation’ (Barnett and Emms 1999: 246). The case of coconut palms in the Chagos Archipelago thus exemplifies the spatial challenge in classifying a species as native or alien according to its natural historical range on such tiny islands: there is general agreement that coconut palms were ‘naturally’ present on the fringes of many islands, yet they were also found further inland on some islands long before the widespread cultivation that resulted in their prevalence in the form of plantations.

## The Barton Point Native Hardwood Restoration Project

Over the past four decades, in the absence of a human population and managed cultivation of the coconut plantations, coconut vegetation has apparently become increasingly dense, except where coconut palms were bulldozed to make way for construction of military base infrastructure (US Naval Facilities Engineering Command (Pacific Division) 1973: 12). Ecologists have bemoaned the uncontrolled overgrowth of the coconut palms on the Chagos islands. Barnett and Emms proposed that Chagos would benefit from:

Control and removal of ‘non-native’ vegetation: The coco palm is the dominant introduced vegetation on many, if not all, of the islands and atolls. This species should be controlled by selective felling to allow regeneration of the native flora. (1999: 246)

Sheppard *et al.* (1999: 15) advocated the spread of ‘native broad-leaved tree species’ – ‘at the expense of the coconut palms’ – on the grounds that it would help ‘to create vegetation more reminiscent of natural, pre-human colonisation conditions.’ Ten years later, Peter Carr, a British military officer on Diego Garcia, put this into practice with the Barton Point Native Hardwood Restoration Project. He reported on the initial stage of the project for *Chagos News*, the periodical newsletter of the CCT, calling for:

… the reversion of the monoculture of coconut *Cocos nucifera* stands back into what stood before the days of the plantations. On all islands where coconut was cultivated as a crop, the relict stands have formed dense, overgrown areas that have become virtually uninhabitable for any other flora. As a result, these anthropogenic suppressors of biodiversity have earned their description of ‘coconut chaos.’ (2010: 16)

Coconut palms are problematic for three reasons. First, living palm fronds prevent light from penetrating below the canopy, and dead palm fronds clog up the ground, preventing other species from regenerating. Second, coconut palms provide shelter and food for rats, a pest unintentionally introduced to the islands by humans. Third, the replacement of coconut palms with indigenous hardwoods would provide extra nesting sites for the native red-footed booby, of which the colony in the Barton Point Reserve is now the largest in the Indian Ocean (and one of the largest in the world). The Restoration Project entailed removing coconut palms, removing coconuts from the ground to prevent reseeding, and replanting hardwoods (Carr 2010: 13–14). In 2011, Carr reported that he had been pleased to discover ‘how quickly the natural recovery of native forest can occur once coconuts are removed’ (Carr 2011: 8).

The shifting classification of coconuts as both native and alien was clarified during the CCT annual conference in 2011. Colin Clubbe stated that the purpose of the Restoration Project is to remove invasive species – such as coconuts and rats – in order to allow native biodiversity to thrive and expand, and entails training residents on Diego Garcia to encourage ‘leaving anything that’s native there.’ After Clubbe’s presentation, the first question from the audience was: ‘Is the coconut non-native?’ Clubbe responded that coconuts are thought to be truly native to a small area of the Pacific, but they moved around the world on their own accord, so are effectively native everywhere; the problem is that they are normally confined to the fringes of islands, where they are an important part of the island ecology, but they pose problems when they are cultivated inland. (This chimes with Robert Scott’s view as quoted above.) The questioner persisted: ‘So coconut was always there?’ Clubbe responded: ‘I suspect so. I don’t really know the origins, but I suspect it’s a natural colony’.

This shifting classification is also evident in Clubbe’s report of his visit to Sea Cow (the smaller of the two Eagle Islands), Moresby, and the Three Brothers:

These islands give us a glimpse of the pristine state of the islands before man and can provide us with clues to the original species composition and structure as we try and restore some of the coconut plantations back to native communities, which will improve the future for the biodiversity of Chagos. (2010: 9)

Clubbe did not consider the coconut plantations to belong to the ‘native communities’ on these islands, but – as we have seen above – both Blair and du Roslan had described the Three Brothers prior to human activity has having been covered in coconut trees. The ‘original species composition’ on these islands evidently included ‘native communities’ of coconut palms on the fringes of the islands and, in some cases at least, further inland. It is not the fringe communities but the formerly cultivated but now overgrown coconut plantations which are perceived as invasive and undesirable and the target of the Restoration Project.

The perception of coconut plantations as invasive – and therefore the *raison d’être* of the Restoration Project – may also be subject to critique. According to the botanical survey conducted by Whistler in 1995, the abandonment of the plantations had enabled other tree species ‘to become established beneath the palms’ (1996: 9). He concluded that:

Eventually, the littoral forest trees will probably take over and coconuts will become a minor component or even disappear. This is what appears to have happened at Minni Minni, which was abandoned long ago. Directly behind the plantation houses is a forest of *Hernandia* with a few old coconuts mixed in, which is all that is left of what was probably once a coconut plantation. (1996: 9)

A 2006 report by the Joint Nature Conservation Committee (JNCC) on the implications of alien and invasive species on Chagos, did not identify coconut as invasive (Varnham 2006: 10). In 2012, Remote Sensing specialists Sarah Hamylton and Holly East described the former plantations of the eastern sector of Diego Garcia as ‘mixed forests’ that had ‘retained a highly diverse proportion of native canopy trees’ (Hamylton and East 2012: 3457). Thus, in addition to being classified as native to some areas but non-native to others, coconut trees are also perceived as invasive in some areas but non-invasive in others.

## Coconut Plantations as Chagossian Cultural Landscapes

The replacement of coconut trees with hardwoods is intended not to improve the environment for human habitation, but to improve the environment for habitation by the native red-footed booby – thus improving biodiversity – while removing a habitat favoured by invasive rats. In defining coconut palms as an invasive alien species to be removed, however, the proponents of the Restoration Project downplay the long history of the coconut plantations on the islands and neglect the social significance of coconuts in the human history of the islands.

Coconut plantations were the economic base and main source of employment on the Chagos islands, and workers received coconut products as part of the rations they received in part-exchange for their labour. Coconuts were a major part of the islanders’ diet: Chagossians extracted the sap to make an alcoholic coconut toddy called *kalu*; they drank the water of unripe coconuts; they made rich *seraz* dishes out of octopus, fish, fowl, green sea turtle, lentils, fruit or vegetables cooked in the milk extracted from ripe coconut flesh; and they used grated coconut flakes to make coconut chutney and sweetmeats such as coconut crunch. Chagossians repeatedly emphasised to me that all parts of the coconut plant could be used, and nothing was thrown away: dried coconut flesh (copra) was pressed to produce coconut oil (for consumption, in cosmetics, and as fuel), and the remaining fibrous copra meal could be used as animal feed; coconut shells could be heated and used for ironing; coconut husks were burned as a cooking fuel, and the ashes could be mixed with coconut oil to produce soap; coir from the husks was made into mattresses and pillows; and coconut fronds were used for roofing, woven into brooms, bags, and baskets, or twisted into rope. In this context, it is hardly surprising that coconut-based cuisine and coconut products feature strongly in Chagossian cultural expression in exile (see Jeffery and Johannessen 2011).

In 2006, the UK Government organised the first ever large-scale return trip to Chagos for 100 islanders, who visited the three main formerly inhabited islands: Diego Garcia, Ile du Coin (Peros Banhos Atoll), and Ile Boddam (Salomon Atoll). When I interviewed them on their return, their main complaint concerned the abandonment and dereliction of their former houses, chapels, cemeteries, clinics, schools, and pathways. They described coconut palms lying over the sea and blocking the routes through the interior of the islands, and overgrown vegetation causing damage to the remnants of the human settlements. Such changes were routinely described as a change from a ‘clean’ (*prop*) to a ‘dirty’ (*sal*) environment because there were no longer people living there and looking after the land (Jeffery 2013: 307–308). Chagossians saw their own relations with the environment in terms of generalised reciprocity: interactions between the inhabitants and their environment were, from their perspective, mutually beneficial. In essence, they argued that when they lived on Chagos they lived in harmony with the environment, took only what they needed, used all parts of a plant or animal, shared any excess so as to reduce waste, and kept their environment neat and tidy by caring for plants and sweeping paths clean.

Return visits to the outer islands since 2006 have revealed the changes in the environment since their departure. From a social perspective these changes were negative: uncontrolled vegetation had become wild and trees had become overgrown, making it difficult to negotiate one’s way around the islands on foot and sometimes making it impossible to find remnants of the former settlements. One of the main tasks undertaken during return visits has been *netwayaz* (cleaning, tidying, weeding): the demarcation of the human presence on the land by cutting down and clearing coconut trees and other overgrown vegetation in the former settlements (Johannessen 2011: 202). A key argument made by Chagossians is that Chagos needs its inhabitants in order to keep its environment in good (i.e., tidy and useable) condition (Johannessen 2011: 205). The *netwayaz* periodically undertaken by visiting Chagossians poignantly serves to simultaneously commemorate the human past and inscribe a human future on the islands, and demonstrating that the coconut plantations comprise a valued cultural landscape.

The Chagossian groups representing the majority of Chagossians opposed the UK Government’s establishment of the MPA and have therefore declined to participate in conservation projects seen to valorise the MPA, but a few Chagossians in favour of the MPA have been involved in the Restoration Project. Allen Vincatassin was a young child when his family was removed from Diego Garcia in 1971. In 2000, Vincatassin established an organisation specifically for Chagossians from Diego Garcia, and in 2011, together with two young men of Chagossian parentage, visited Diego Garcia on what he described as a ‘conservation mission.’ Vincatassin told me that ‘coconuts have invaded all the areas. The Barton Point Project is about removing invasive species and bringing endemic plants back so there is a balance’. Here Vincatassin had taken on board the stated aims of the Restoration Project (except for that he referred to ‘native’ plants as ‘endemic’). He then added a social dimension – more reflective of the statements made by other Chagossians – that the clearing of the land will make the area ‘more alive: we will be able to see the old houses which we can’t see now because of the forest, and in three years people will say “that’s the plantation I knew”.’ Older Chagos islanders – including those opposed to the MPA – similarly expressed their approval of the removal of overgrown coconut trees, which formed part of the weeding (*labati*) they had carried out during the plantation era (see Wiehe 1939: 18), and also noted that the removal of overgrown trees would make the islands more accessible for humans in future. Thus Chagossians and conservationists seem to have converged in their argument that the overgrown coconut plantations need to be controlled, albeit for different reasons.

## Conclusion: Restoration Ecology in Cultural Landscapes

Restoration ecology is less about the removal of alien species per se than it is about the removal of harmful invasive species that threaten biodiversity (Milton 2000; Helmreich 2005; Simberloff 2003; Warren 2007). Invasiveness is a slippery category, however, since the resilience and spread of a species is influenced by anthropogenic and environmental factors and it is thus tricky to predict the lasting impacts of a species within an ecosystem over time (Richardson and Pyšek 2006). The removal of an invasive species can be controversial when it also has economic or cultural value, resulting in disputes between ecologists and local people (Comaroff and Comaroff 2001).

It is not radical to suggest that planned ecological restoration projects should consider the social and economic values attributed to species by people such as local land users, but my research suggests that conservationists and Chagossians share the view that the coconut plantations have become overgrown and need to be controlled. There remain, however, key differences in their approaches. For the conservationists I interviewed, coconut coverage should be *reduced* for ecological reasons: a return to an imagined pre-human environment in which biodiversity flourishes. For the Chagossians interviewed, the coconut coverage should be *managed* for social reasons: firstly to make visible the human history on the islands, secondly to improve the accessibility of the islands for humans, and thirdly in preparation for the potential reopening of the plantations in the event of resettlement.

The Chagos case is also characterised by a widely disparate balance of power: conservation groups managed to persuade the UK Government to introduce a no-take MPA, while the displaced Chagossian community is chronically disadvantaged and has historically been marginalised in decision-making about the future of the Chagos Archipelago. One of the major stumbling blocks in working towards a sustainable future for the Chagos Archipelago is that the conservationist and Chagossian communities have increasingly become pitted against each other, not least because exclusionist conservationists fear that resettlement would be detrimental to the ‘pristine’ Chagos environment, and because many Chagossians believe that the MPA was introduced in order to prevent them from returning to Chagos. The comparatively powerful conservationist community could reach out to the disadvantaged Chagossian community through dialogue to determine if the two groups can agree on mutually acceptable levels of coconut tree removal and coconut plantation management. One solution that could reconcile ecological restoration with the restoration of cultural landscapes would be to reduce or remove some of the former coconut plantations in pursuit of the laudable conservation goals discussed above, while preserving and managing other former coconut plantations in recognition of the Chagossians’ historical presence and potential future on Chagos.
